# TraN variants mediate conjugation species specificity of IncA/C, IncH, and *Acinetobacter baumannii* plasmids

**DOI:** 10.1128/jb.00536-25

**Published:** 2026-03-19

**Authors:** Shan He, Sophia David, Jaie Rattle, Julia Sanchez-Garrido, Wen Wen Low, Joshua L. C. Wong, Konstantinos Beis, Gad Frankel

**Affiliations:** 1 Department of Life Sciences, Imperial College London 98455, London, United Kingdom; 2 Centre for Genomic Pathogen Surveillance, Pandemic Sciences Institute, University of Oxford 685411, Oxford, United Kingdom; University of Virginia School of Medicine, Charlottesville, Virginia, USA

**Keywords:** antimicrobial resistance, IncA/C and IncH plasmids, bioinformatics, plasmid conjugation, *Klebsiella pneumoniae*, *Escherichia coli*, *Citrobacter*, horizontal gene transfer

## Abstract

IncA/C and IncH plasmids commonly carry antimicrobial resistance genes, notably *bla*_NDM-1_. Although these plasmids disseminate among Gram-negative pathogens via conjugation, the mechanisms underlying mating pair stabilization (MPS) and conjugation species specificity remain poorly understood. In IncF plasmids, MPS is mediated by interactions between outer membrane proteins (OMP) encoded by the plasmids in the donor (TraN) and by the chromosome in the recipient. Using the Plascad database, we extracted 1,436 TraN sequences from 1,517 plasmids: 62.5% (898/1,436), mainly in IncF plasmids, are 550–660 amino acids (aa) (we renamed TraN short, TraN_S_); 15% (216/1,436), in IncA/C plasmids, are 880–950 aa (TraN medium, TraN_M_); and 11% (160/1,436), in IncH plasmids, are 1,050–1,070 aa (TraN long, TraN_L_). One TraN, found in six plasmids from *Acinetobacter baumannii* (891 aa), was designated TraN V-shaped (TraN_V_). Like TraN_S_, TraN_M_ and TraN_L_ contain a base and one distal tip domain essential for conjugation, whereas TraN_V_ has a base and two distinct tip domains forming a V-shaped structure. TraN_M_, TraN_L,_ and TraN_V_ determine conjugation species specificity, with TraN_L_ cooperating with OmpA. Tip swapping reverses conjugation specificity, revealing how TraN_M_ and TraN_L_ diversity influence plasmid host range and AMR dissemination. Our new data reveal the molecular basis of plasmid host specificity and broaden our understanding of how conjugation drives the dissemination of antimicrobial resistance genes among clinically relevant bacteria.

**IMPORTANCE:**

Plasmid conjugation drives the spread of antimicrobial resistance genes between different bacterial species. In IncF plasmids, this process relies on tight interactions between an outer-membrane protein in the recipient and the plasmid-encoded TraN, which consists of conserved base and variable tip domains. So far, TraN was only studied in IncF plasmids. We show that IncA/C and IncH plasmids encode a larger TraN with distinct isoforms that shape host range and species specificity. We also identify a novel TraN variant in *Acinetobacter baumannii* plasmids containing a base and two tips. These findings broaden our understanding of conjugation specificity and the mechanisms that influence the dissemination of resistance genes across diverse bacterial communities and highlight the evolutionary flexibility of plasmid transfer systems.

## INTRODUCTION

Infectious diseases pose a continuous and increasing threat to global public health (1). The rapid spread of antimicrobial resistance genes (ARGs) amongst pathogens is affecting our ability to treat many acute bacterial infections, including those with important broad-spectrum antimicrobials, such as carbapenems, used for severe infections (2, 3).

The spread of ARGs is facilitated by diverse mechanisms of horizontal gene transfer (4, 5), among which conjugation is the major driver (6). During conjugation, a plasmid is transferred unidirectionally from a donor bacterium to a recipient bacterium in a contact-dependent manner (7, 8). Conjugative plasmids can disseminate within and between species, affecting bacterial evolution. Successful plasmid conjugation and maintenance in the recipient is a process involving multiple steps. First, the plasmid-encoded mating pilus bridges the donor with a potential recipient (7). In IncF plasmids, this is followed by the formation of a donor-recipient synapse (8). Finally, entry of the leading single-stranded plasmid DNA activates defense responses in the recipient (9), which, if counteracted by plasmid-encoded anti-defence systems, results in the formation of transconjugants (10). This is followed by the expression of plasmid genes encoding surface and entry exclusion (11, 12).

DNA transfer is facilitated by a type IV secretion system (T4SS) (13) and the attached mating pilus. Binding of the pilus expressed by the donor to a recipient leads to mating pair formation (MPF). Based on homology between the components of the T4SS and additional accessory factors that are involved in MPF, the conjugation systems have been classified into four groups—MPFG, MPFT, MPFI, and MPFF (14, 15). MPFF plasmids, which express long conjugative pili and belong to the IncF, IncH, and IncA/C groups, account for more than a third of conjugative plasmids isolated from Gammaproteobacteria and are particularly highly represented amongst Enterobacterales species (16).

The reference IncHI1 plasmid, R27, was isolated from *Salmonella enterica* serovar Typhi in 1961 (17). IncHI1 and IncHI2 plasmids are thermosensitive for transfer with an optimum rate of transfer between 22°C and 30°C (18). A mutation in *htdA* derepresses R27 (drR27), resulting in constitutive expression of the conjugation machinery and the H pilus (19). The reference IncA/C plasmid, RA1, was isolated from *Aeromonas liquefaciens* in 1971 (20). RA1 expresses the conjugation machinery constitutively at 30°C (21).

The cryo-EM structures of the mating pili expressed by the IncF-like plasmids pOX38, pED208, and pKpQIL revealed that the linear TraA pilin subunits form helical assemblies with phosphatidylglycerol (PG) molecules at a stoichiometric ratio of 1:1 (22). More recently, we reported that the H pilus, encoded by R27, is an assembly of PG-associated cyclic TrhA pilin subunits (23). Pilus-mediated MPF enables inefficient DNA transfer from a distance (24, 25). Formation of MPF is followed by pilus retraction, leading the donor and recipient bacteria forming mating junctions (26), characterized by intimate wall-to-wall contact, through a process termed mating pair stabilization (MPS) (27), which mediates efficient DNA transfer (28). We have reported that in IncF-like plasmids, MPS is mediated by interactions between one of four plasmid-encoded TraN isotypes in the donor, classified as TraNα, TraNβ, TraNγ, and TraNδ, specifically with OmpW, OmpK36, OmpA, and OmpF in the recipient, respectively (28, 29).

AlphaFold3 structural prediction of the different TraN isotypes encoded by IncF-like plasmids reveals that they consist of a conserved base domain, predicted to embed the protein in the donor outer membrane, and a variable distal tip domain, which mediates conjugation efficiency, specificity, and host range (30). We have shown that while TraNα and TraNδ mediate broad conjugation host range, TraNβ and TraNγ mediate narrow conjugation host range as they are mainly found in *Klebsiella* species and in *Escherichia coli,* respectively (28).

While IncA/C and IncH plasmids also belong to the MPFF group and encode TraN, less is known about how they establish donor-recipient interactions and MPS, even though they are important vehicles for the spread of antibiotic resistance in *Escherichia coli, Klebsiella pneumoniae,* and *Acinetobacter baumannii*, particularly the carbapenemase New Delhi metallo-β-lactamase-1 (NDM-1) (31).

In this study, we characterized the IncA/C and IncH plasmid-encoded TraN, as well as TraN encoded by plasmids found exclusively in *A. baumannii*. As these TraN proteins are ca. 50% larger than IncF plasmids-encoded TraN, which we have now reclassified as TraN short (TraN_S_), we classified the IncA/C-encoded TraN as TraN medium (TraN_M_), the IncH-encoded TraN as TraN long (TraN_L_), and the *A. baumannii-*encoded TraN as TraN V-shaped (TraN_V_), respectively. Using bioinformatic, structural, and conjugation analyses, we reveal their diversity, host range preferences, and receptor dependencies. Together, these findings provide new insights into the molecular basis of plasmid host specificity and broaden our understanding of how conjugation drives the dissemination of antimicrobial resistance among clinically relevant bacteria.

## RESULTS

### Identification of TraN homologs in IncA/C and IncH plasmids

We searched for TraN protein sequences among 1,517 MPFF plasmids in the Plascad database (32), using the sequence annotations. 89.5% (1,358/1,517) of the plasmids contained a single TraN, and 2.5% (38/1,517) contained 2–3 copies. No TraN was found in 8.0% (121/1,517) of the plasmids. Of the 121 plasmids with no annotated TraN, these were largely from Enterobacteriaceae species (73.6%; 89/121) and carried a variety of replicon types, including IncF subtypes (60.3%; 73/121) and IncA/C2 (15.7%; 19/121), while 20.7% (25/121) carried no known replicon type. Altogether, we extracted 1,436 TraN protein sequences from the 1,517 plasmids.

The length of the identified TraN proteins was highly variable; 62.5% (898/1,436) were 550–660 amino acids (aa) long, which we have now renamed TraN short (TraN_S_), with many of these derived from plasmids carrying IncF replicons and carried by Enterobacterales species. We also noted additional distinct groupings based on size, with a further 15.0% (216/1,436) possessing a length of 880–950 aa, which we classified TraN medium (TraN_M_), and 11.1% (160/1,436) constituting 1,050–1,070 aa, which we classified TraN long (TraN_L_). Other smaller groups include six identical TraN of 891 aa from *A. baumannii* plasmids, which we classified as TraN V-shaped (TraN_v_), nine TraN of 1,882 aa from different *Xanthomonas* species, and nine TraN of 885–931 aa from different Enterobacterales species. Representative plasmids encoding TraN_S_, TraN_M_, TraN_L_, and TraN_V_ (including the proteins’ ID and tip domains) are listed in Table 1.

**TABLE 1:** List of TraN proteins

TraN	Plasmid	Protein ID	Protein length	Tip residues
TraN_M_α	RA1	ACN66968.1	912	230C-360D
TraN_M_β	pNDM-US	AHI38890.1	932	205N-354V
TraN_L_α	R27	AAF69844.1	1,058	305S-457E
TraN_L_β	pHNAH67	APZ78090.1	1,062	305T-457E
TraN_L_γ	pNDM-MAR	AFB82831.1	1,061	304S-457D
TraN_V_	ABAY10001	QBN23304.1	891	149V-628R

**Fig 1:**
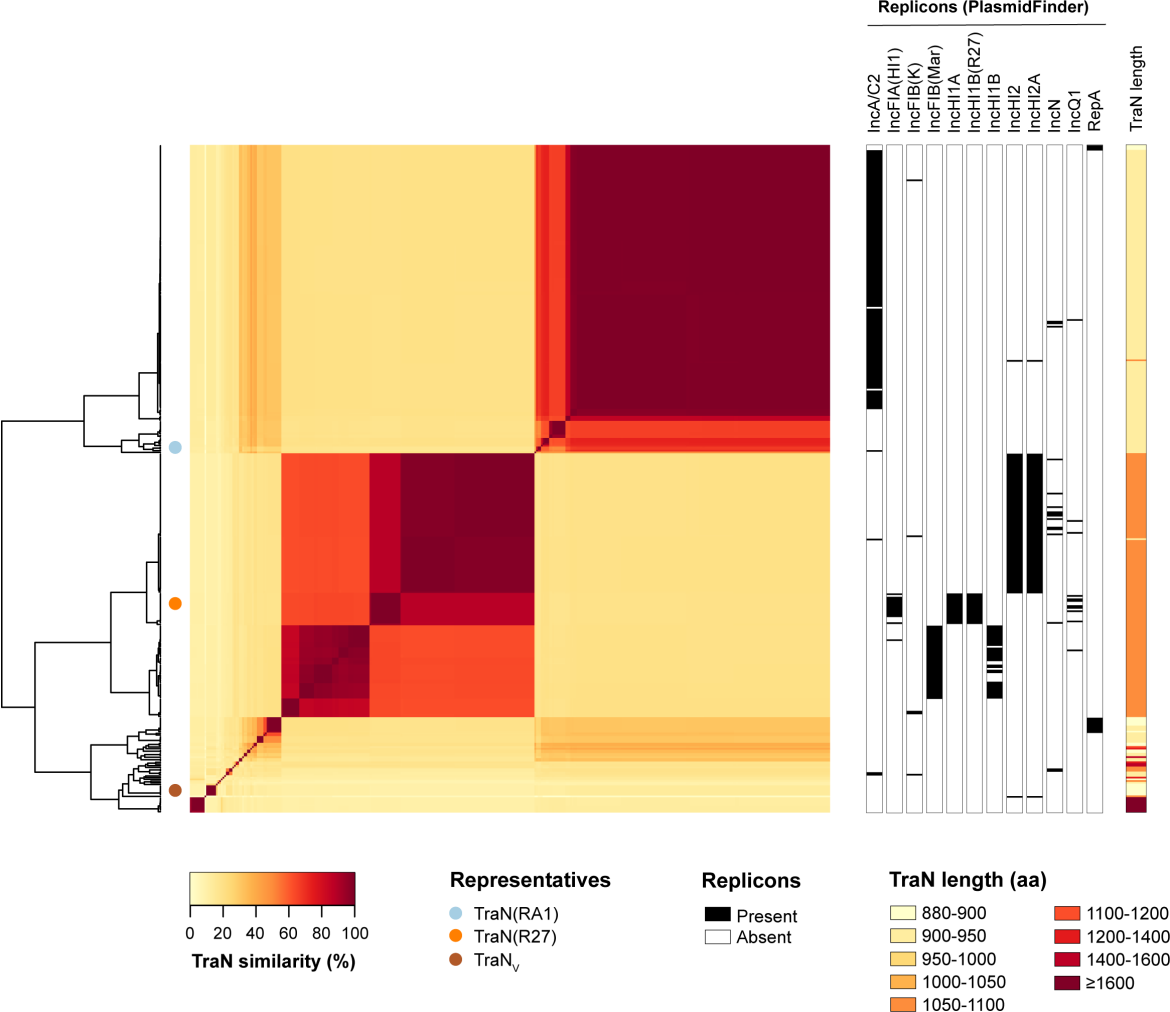
Pairwise similarity of TraN proteins. Heatmap and associated dendrogram showing the pairwise similarity among 392 TraN protein sequences (≥880 aa). Metadata columns show the presence/absence of particular plasmid replicons from PlasmidFinder (those found in ≥5 plasmids) among the associated plasmids and the TraN protein length (aa). The TraN(RA1) and TraN(R27) sequences, as well as a representative TraN_V_ sequence, are highlighted by the filled circles adjacent to the dendrogram.

We compared the pairwise sequence similarity of the 400 TraN proteins ≥880 aa (Fig. 1). A heatmap showing these pairwise similarities illustrates the high level of sequence diversity found among these proteins overall, with similarities as low as 10% between some pairs. However, two major groups, which can be further subdivided into subgroups, were observed based on protein similarity (Fig. 1). The first major group comprises TraN_M_, which is largely associated with plasmids carrying either an IncA/C (2/184) or IncA/C2 replicon (151/184), including the prototypical IncA/C plasmid RA1. All associated plasmids from this TraN group are of mob type MOBH (184/184) and mostly belong to the plasmid taxonomic unit (PTU), PTU-C (153/184), with the remaining plasmids unassignable to a PTU. The other major group, TraN_L_, is largely associated with plasmids encoding different IncH replicons (135/155), and includes the prototypical plasmid R27, which encodes IncHI1A, IncHI1B(R27), and IncFIA(HI1) replicons. All but one of the associated plasmids are categorized as MOBH (154/155) and categorized into five different PTUs (with a further seven plasmids where the PTU could not be assigned). The other smaller groups represented in the heatmap include TraN_V_ (Fig. 1), which is found in plasmids belonging to the MOBF type (6/6) and PTU-Pse1 (6/6).

### Distribution of TraN_M_ and TraN_L_ in host taxa

**Fig 2:**
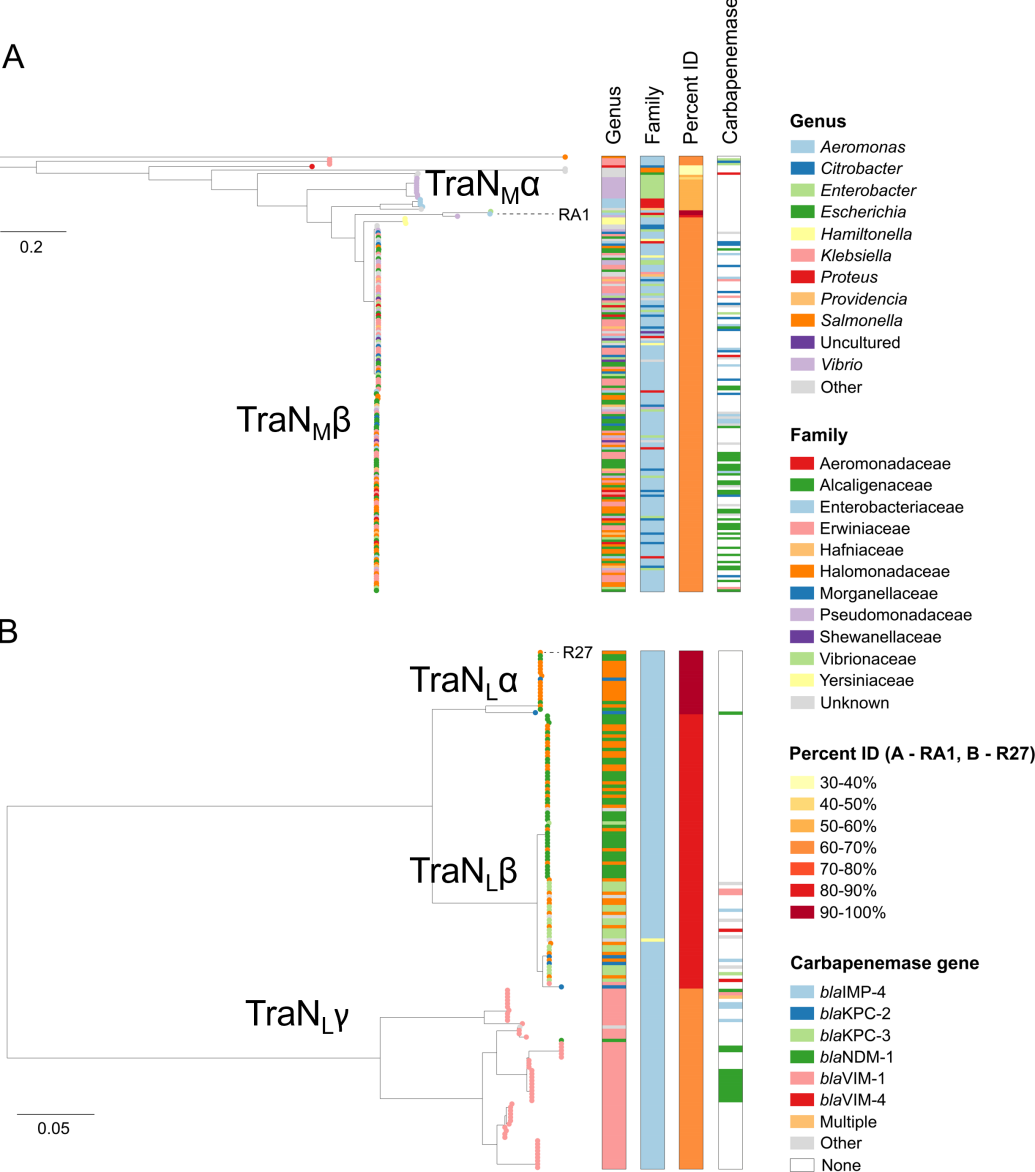
Phylogenetic analysis of TraN proteins. Phylogenetic trees of TraN protein sequences (≥880 aa) with ≥30% similarity to TraN(RA1) (**A**) and TraN(R27) (**B**). The trees contain 184 and 155 sequences, respectively, and are midpoint-rooted. The tree tips show the host genus of the associated plasmid. Metadata columns show the host genus and family of the associated plasmid, the percent identity of each TraN variant with respect to TraN(RA1) (**A**) or TraN(R27) (**B**), and the presence of carbapenemase gene variants within the plasmid. The scale bars show the number of substitutions per site. Similar visualizations can be accessed via Microreact: https://microreact.org/project/tran-ra1 (**A**) and https://microreact.org/project/tran-r27 (**B**).

To investigate TraN_M_ and TraN_L_ relatedness at high resolution and their association with host taxa, we constructed two maximum-likelihood phylogenies of the 184 and 155 TraN proteins with ≥30% similarity to RA1- and R27-encoded TraN, respectively. This revealed that while a minority of TraN_M_ belong to diverse lineages, TraN_M_α, most TraN_M_ belong to a major dominant clade, TraN_M_β. The TraN_M_ proteins represented in the tree were obtained from plasmids derived from at least 11 different host families, with Enterobacterales accounting for 66.3% (122/184). TraN_M_ variants from different families (and different genera) were highly interspersed within the tree, suggestive of frequent exchange of the associated plasmids between these host taxa (Fig. 2A; https://microreact.org/project/tran-ra1, which includes plasmid typing data).

The TraN_L_ group can be divided into three clades, which we named TraN_L_α, which includes R27, TraN_L_β, and TraN_L_γ. Notably, all but one of the TraN_L_ proteins are from plasmids found in Enterobacterales. Of note, we observed clear associations of clades with one or more genera. Plasmids encoding TraN_L_α are mainly found in *Salmonella*/*Escherichia*, TraN_L_β is mainly found in *Salmonella*/*Enterobacter,* and plasmids encoding TraN_L_γ are almost exclusively found in *Klebsiella* (Fig. 2B; https://microreact.org/project/tran-r27, which includes plasmid typing data).

Importantly, 39.1% (72/184) and 15.5% (24/155) of the plasmids encoding TraN_M_ and TraN_L_ carry a carbapenemase gene, respectively, with a particular predominance of *bla*_NDM-1_ (Fig. 2). Taken together, these findings suggest plasmids expressing TraN_M_ are major vectors of *bla*_NDM-1_ and exhibit a broad host range, while a smaller proportion of plasmids expressing TraN_L_ encode *bla*_NDM-1_ and show a high degree of host restriction.

### TraN_S_, TraN_M_, and TraN_L_ are essential for conjugation

**Fig 3:**
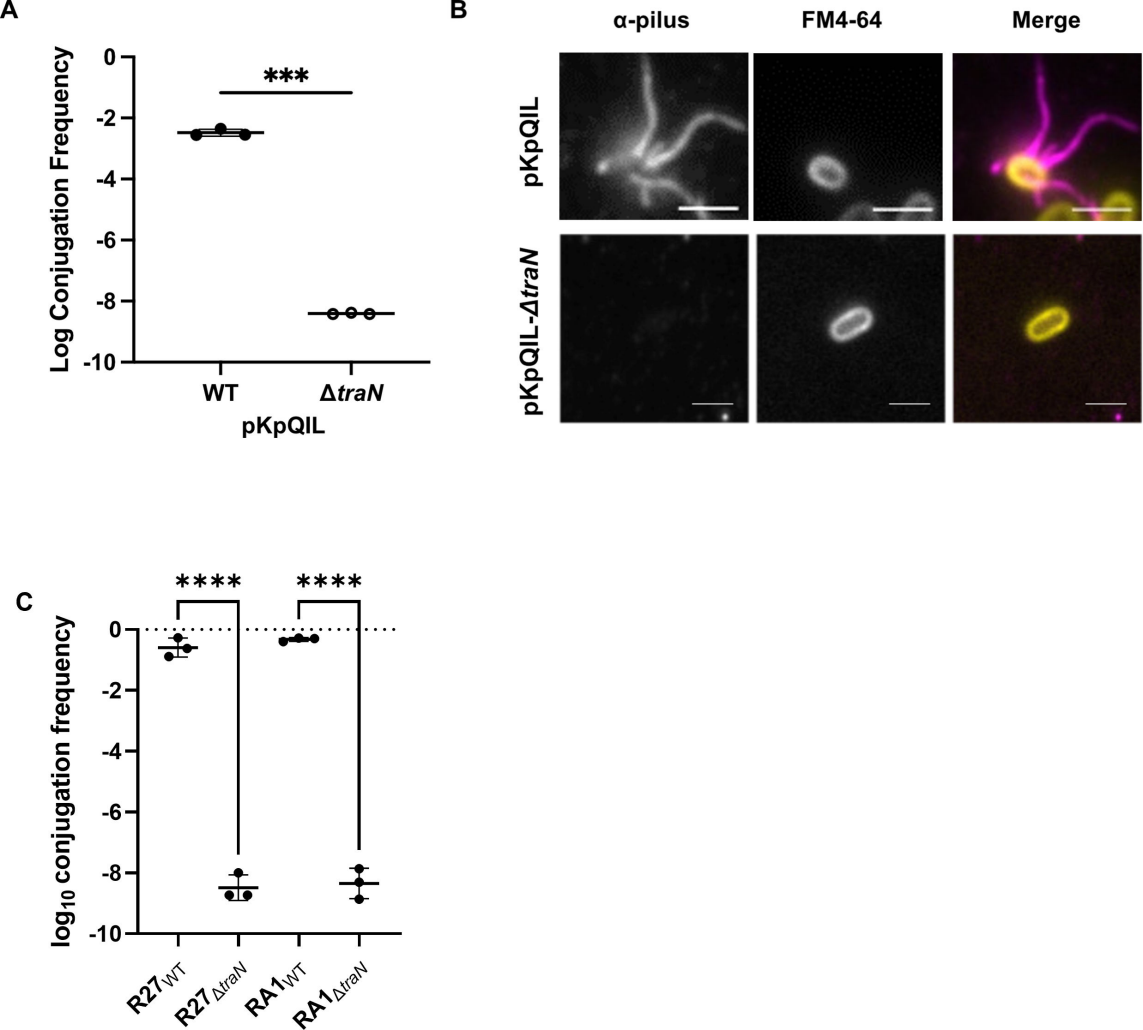
Deletion of *traN* abolishes plasmid transfer and donor cell piliation. (**A**) Log conjugation frequency of wild-type pKpQIL and pKpQILΔ*traN_S_*. Open circles indicate that transconjugant counts were below the limit of detection. Data analyzed by paired *t*-test. ***, *P* < 0.001; ****, *P* < 0.0001. Error bars represent SD (*n* = 3). (**B**) Immunofluorescence staining of the maiting pili on pKpQIL and pKpQILΔ*traN_S_* donor bacteria. Scale bar = 2 µm. (**C**) Log conjugation frequency of wild-type R27 and R27Δ*traN_L_* and wild-type RA1 and RA1Δ*traN_M_*.

Previous observations showed that deletion of *traN* in pED208 did not completely interfere with pilus biogenesis and function. However, while the mutant-mediated transfer, it was significantly less efficient than the wild-type (WT) plasmid (33). To investigate the role of TraN_S_, TraN_M_, and TraN_L_, we generated TraN-deficient pKpQIL, RA1, and R27 plasmids. All three plasmids lacking TraN were conjugated at an efficiency below the level of detection (Fig. 3A and C). We then used our polyclonal pKpQIL pilus antiserum to investigate if TraN_S_ plays a role in pilus biogenesis. This revealed that while mating pili were elaborated on donors containing wild-type pKpQIL, no pili were seen on donors containing pKpQIL *ΔtraN_S_* (Fig. 3B). This suggests that, in addition to playing a role in MPS, TraN also impacts pilus biogenesis.

### TraN_M_ and TraN_L_-mediated recipient conjugation specificity

**Fig 4:**
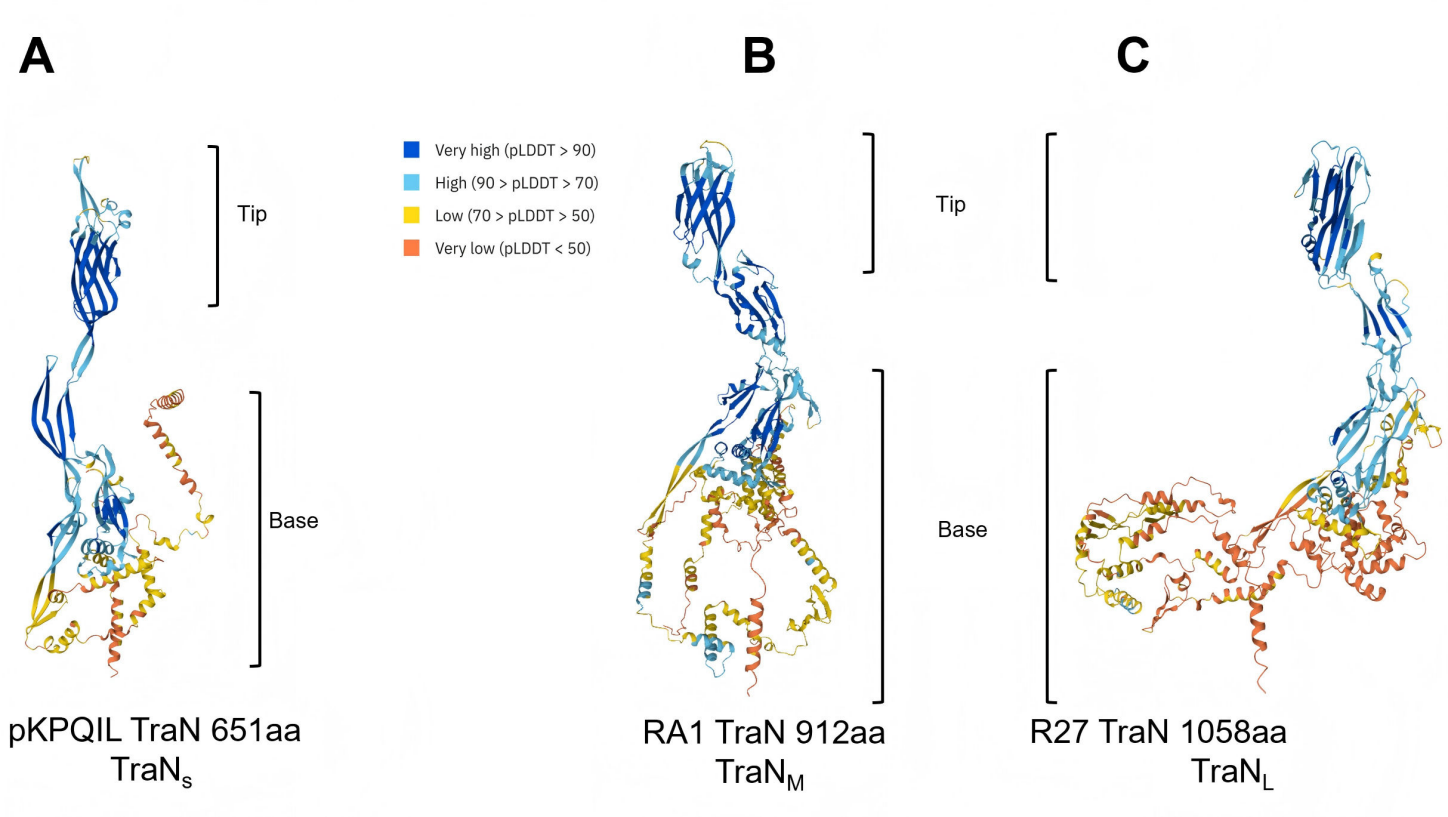
AlphaFold3 prediction and conjugation specificity of R27 and RA1. AlphaFold3 structural prediction of TraN encoded by pKpQIL (**A**), RA1 (**B**), and R27 (**C**). The predicted local distance difference test (pLDDT) is a per-residue measure of local confidence, scaled from 0 to 100.

We have shown before that the tips of the four TraN_S_ variants (TraN_S_ α, β, γ, δ) (Fig. 4A), mediate MPS, conjugation efficiency, and species specificity (28). AlphaFold3 structural predictions of RA1 TraN_M_ and R27 TraN_L_ indicate that their architectures can also be divided into base and tip domains (Fig. 4B and C). Compared with the TraN_S_ family, these proteins exhibit substantial size and structural variability (Fig. 4B and C). The base domains contain additional α-helices, whereas the tip regions are dominated by a β-sandwich domain flanked by loops. This contrasts with the TraN_S_ family, whose tip domains contain additional tertiary structural elements.

**Fig 5:**
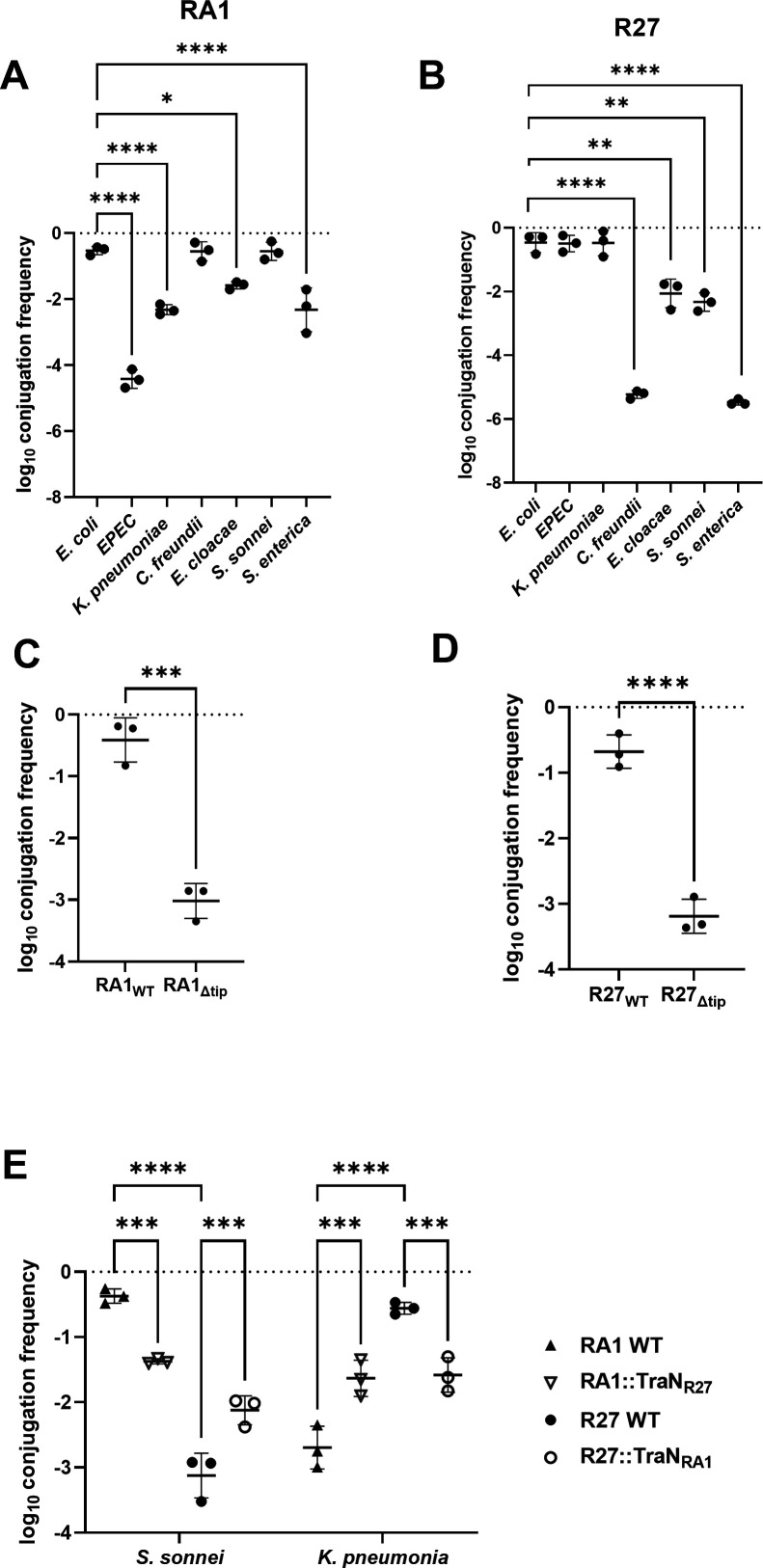
TraN_M_ and TraN_L_-mediated recipient conjugation specificity. Conjugation of RA1 (**A**) and R27 (**B**) into different *Enterobacterales and E. coli* MG1655. Log conjugation frequency data are presented as mean ± s.d. of three biological repeats, analyzed by repeated measures one-way ANOVA with Tukey’s multiple comparison test. Tip-less RA1 (**C**) and R27 (**D**) Log conjugation frequency data are presented as mean ± s.d. of three biological repeats, analyzed by a two-sided paired *t*-test. (**E**) TraN swaps between RA1 and R27 reverse conjugation specificity into *S. sonnei* and *K. pneumoniae* recipients. All the data have been log transformed and presented as mean ± s.d. of three biological repeats, analyzed by repeated measures two-way ANOVA with Tukey’s multiple comparison test. *, *P* < 0.05; **, *P* < 0.01; ***, *P* < 0.001; ****, *P* < 0.0001.

We used *E. coli* MG1655 containing either R27 or RA1 as donors and six well-characterized pathogenic species from the *Enterobacterales* family as recipients in conjugation assays. MG1655 was also used as a recipient control. In agreement with the observed distribution of TraN_M_ in clinical isolates (Fig. 2), RA1 was conjugated efficiently into *Citrobacter freundii (C. freundii), Shigella sonnei (S. sonnei*), and MG1655. Conversely, RA1 was conjugated with lower efficiency (~80-fold lower) into *K. pneumoniae*, *Enterobacter cloacae (E. cloacae*), and *Salmonella enterica* serovar Enteritidis (*S. enteritidis*) and with a very low frequency into enteropathogenic *E. coli* (EPEC, strain E2348/69) (Fig. 5A). R27 plasmid was conjugated with a high frequency into *K. pneumoniae*, EPEC, and MG1655, and with a lower frequency into *E. cloacae* and *S. sonnei* (Fig. 5B). The conjugation frequency dropped significantly when *S*. Enteritidis and *C. freundii* were used as recipients. We have previously shown that the tip of TraN_S_ provides species specificity. Based on this, we sought to establish if the tips of R27 and RA1 also mediate distinct conjugation species specificity among Enterobacterales. To this end, we first generated RA1 and R27 tip-less mutants (Δtip) to be used as negative controls. Quantifying conjugation frequency into MG1655 revealed that RA1 and R27 Δtip were conjugated at ca. 350-fold lower efficiency compared to the corresponding WT plasmids (Fig. 5C through E). We then swapped the tips between RA1 and R27 and quantified conjugation frequencies into *K. pneumoniae* and *S. sonnei* recipients as representatives. This revealed that compared to R27, the R27::TraN_RA1_ was now conjugated with lower efficiency into *K. pneumoniae* and higher efficiency into *S. sonnei*. Conversely, compared to RA1, the RA1::TraN_R27_ conjugated with higher efficiency into *K. pneumoniae* and lower efficiency into *S. sonnei* (Fig. 5E).

**Fig 6:**
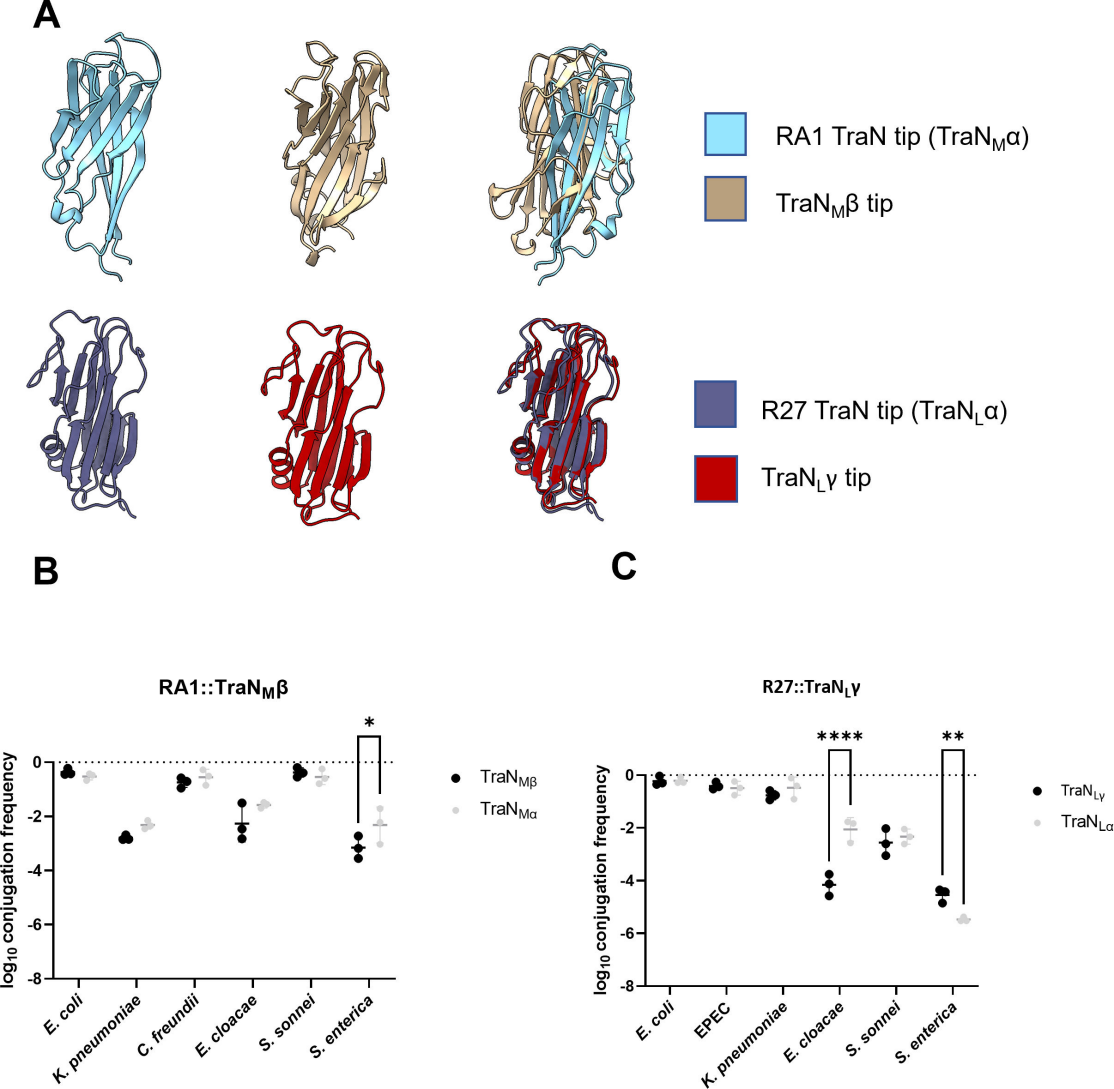
Conjugation specificity of TraN_M_β and TraN_L_γ. (**A**) Cartoon representation of the AlphaFold3 structural prediction of the TraN variant tips. The TraN_M_ and TraN_L_ subfamilies display structural similarities between them. Comparison of conjugation species specificity between TraN_M_α and TraN_M_β (**B**) and TraN_L_α and TraN_L_γ (**C**). The comparison was performed between the TraN variant and its prototypical TraN, and the data have been log transformed and presented as mean ± s.d. of three biological repeats, analyzed by repeated measures two-way ANOVA with Tukey’s multiple comparison test. ns, non-significant; *, *P* < 0.05; **, *P* < 0.01; ***, *P* < 0.001; ****, *P* < 0.0001.

We also tested if the TraN_M_ and TraN_L_ variants seen in the phylogenetic tree (Fig. 2) affected conjugation species specificity. To this end, we selected two resistance plasmids, pNDM-US (34), encoding TraN_M_β and pNDM-MAR (35), encoding TraN_L_γ (Fig. 2). AlphaFold3 structural prediction showed that the structure of the TraN_L_ tips encoded by R27 and pNDM-MAR is very similar despite variations in their sequences (Fig. 6A). In contrast, the TraN_M_ tips encoded by RA1 and pNDM-US showed different folds, particularly at the distal loops (Fig. 6A).

Our panel of Enterobacterales species as recipients revealed that, despite the structural differences, the TraN_M_β tip mediated similar conjugation efficiency as the TraN_M_α tip, except for a subtle decrease of conjugation into the *S.* Enteritidis recipient (Fig. 6B). In contrast, while structurally conserved, R27 expressing the TraN_L_γ tip mediated lower conjugation efficiency specifically into the *E. cloacae* recipient and higher conjugation frequency into the *S.* Enteritidis recipient compared with TraN_L_α; conjugation efficiency into other recipients did not change (Fig. 6C).

Taken together, these results show that the TraN_M_ and TraN_L_ tips mediate MPS and recipient conjugation specificity like the TraN_S_ (28). Despite the high degree of structural similarity between the TraN_L_ variants, subtle sequence changes affect conjugation efficiency into specific recipients.

### TraN_V_ mediates conjugation into *A. baumannii* and selected Enterobacterales species

**Fig 7:**
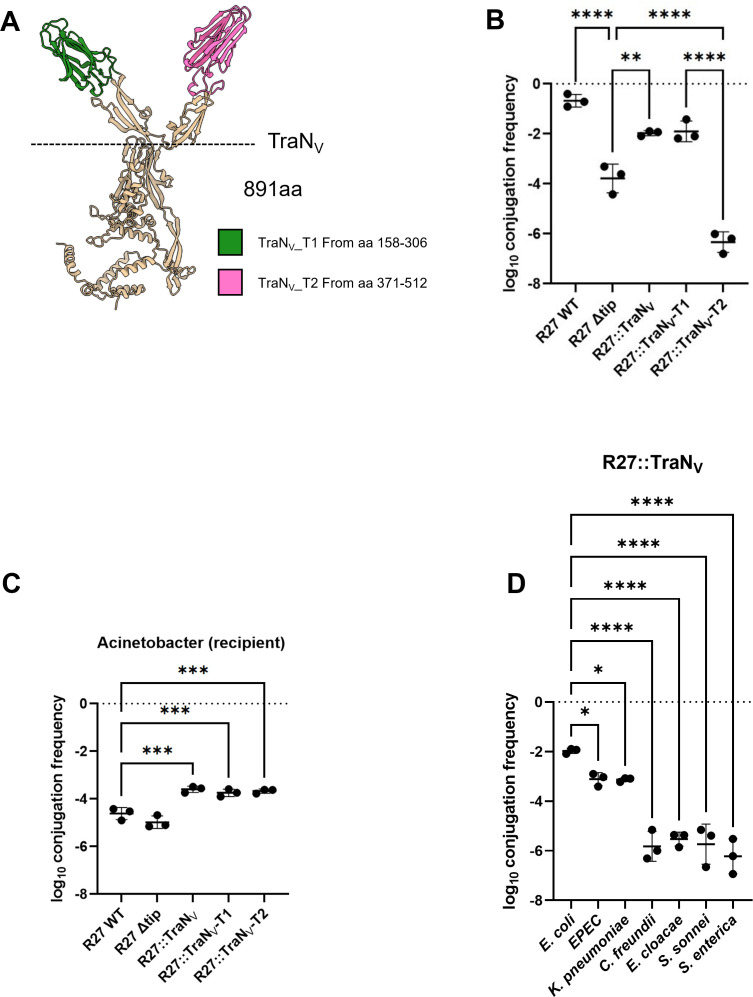
Characterization of TraN_V_. AlphaFold3 structural prediction of TraN_V_. TraN_V__Tip 1 and TraN_V__Tip 2 are shown in green and pink colors, respectively. The dashed line marks the V-shape 2 tips region that was transplanted onto R27 (**A**). Conjugation of TraN_V_ and its respective tips into *E. coli* MG1655 recipient (**B**). Conjugation of TraN_V_ and its respective tips from *E. coli* donor to *A. baumannii* recipient, using WT and Δtip R27 as controls (**C**). Conjugation species specificity of TraN_V_, transplanted onto the R27 TraN_L_ base (**D**). All the data have been log-transformed and presented as mean ± s.d. of three biological repeats, analyzed by repeated-measures one-way ANOVA with Tukey’s multiple comparison test using R27 WT as a control. *, *P* < 0.05; **, *P* < 0.01; ***, *P* < 0.001, ****, *P* < 0.0001.

We identified six 891 aa TraN-encoding plasmids in *A. baumannii* (Fig. 1), whose AlphaFold3 structural prediction revealed a unique architecture of a base domain presenting two structurally distinct tips, arranged into a V-shape; we named them TraN_V_ and annotated the respective tips as T1 and T2 (Fig. 7A). To investigate the role TraN_V_ plays in conjugation, we “transplanted” the V-shaped tip and individually T1 and T2 from pABAY10001, an *A. baumannii* 54 kb plasmid containing no antibiotic resistance genes (GenBank: MK386682.1), onto the R27 TraN base.

We first used MG1655 containing these chimeric TraN variants as donors and MG1655 and an *A. baumannii* strain (ATCC17978) as recipients. MG1655 donors containing R27 or R27∆tip were used as controls (Fig. 7B). R27::TraN_V_ and R27::TraN_V_-T1 were conjugated with similar efficiencies into MG1655, intermediate between R27 and R27∆tip. In contrast, R27::TraN_V_-T2 was not conjugated (Fig. 7B). In contrast, R27::TraN_V_, R27::TraN_V_-T1, and R27::TraN_V_-T2 were conjugated with similar efficiencies into *A. baumannii*, which were higher than both R27 and R27∆tip (Fig. 7C). This suggests that while TraN_L_ does not mediate conjugation into *A. baumannii*, TraN_V_-T1 is the dominant tip during TraN_V_-mediated conjugation into *E. coli*. In contrast, TraN_V_, TraN_V_-T1, and TraN_V_-T2 are equally functional in conjugation into an *A. baumannii* recipient.

Finally, we investigated the conjugation host range mediated by R27::TraN_V_ using our Enterobacterales panel as recipients. This revealed that R27:TraN_V_ was conjugated in high efficiency into MG1655, EPEC, and *K. pneumoniae,* and with much lower efficiency into *C. freundii*, *E. cloacae*, *S. sonnei*, and *S.* Enterica (Fig. 7D).

### TraN_L_-mediated conjugation is dependent on OmpA

**Fig 8:**
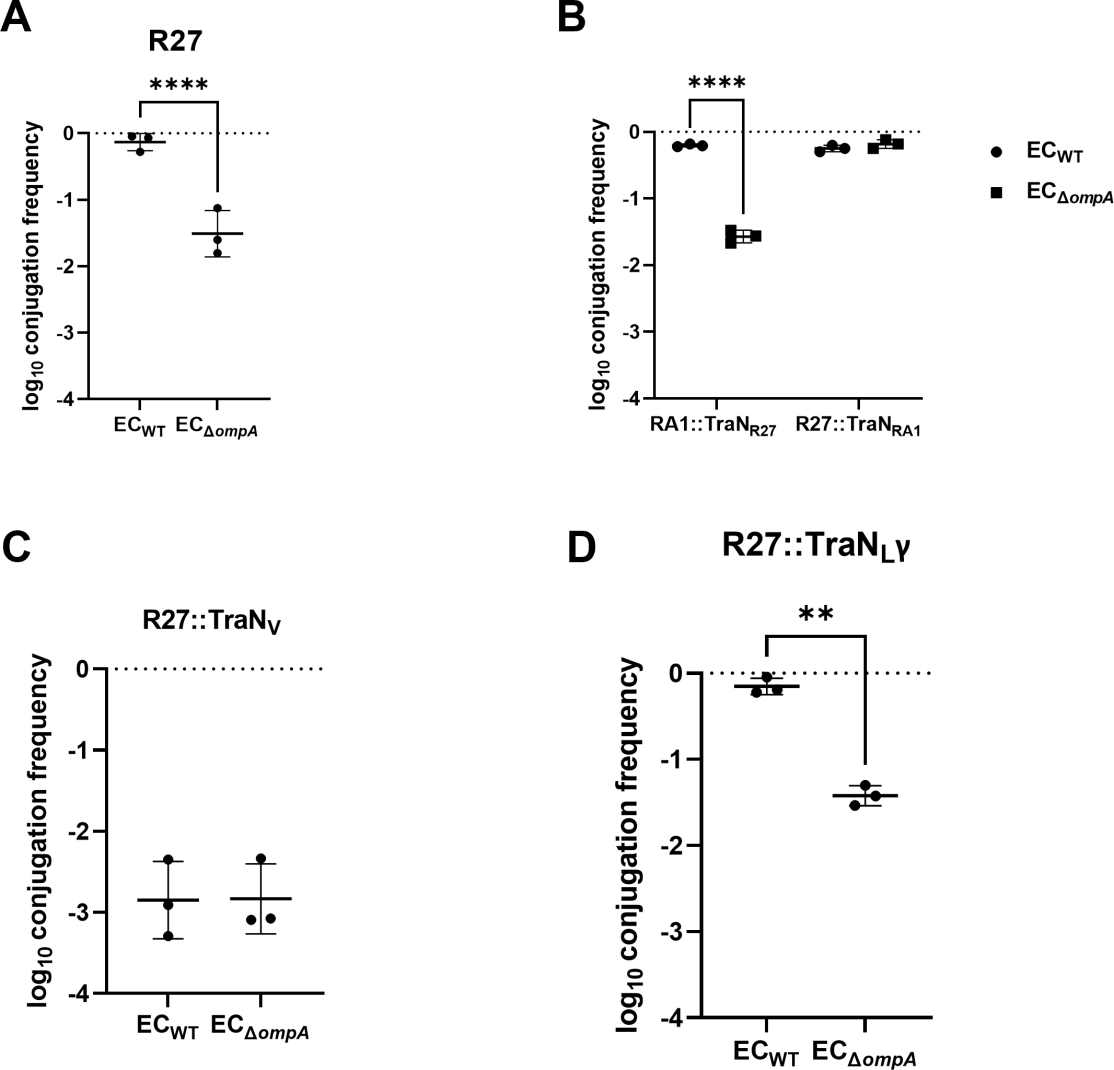
Conjugation of TraN_L_ is OmpA-dependent. Conjugation of R27 is OmpA-dependent (**A**). Swapping tips between RA1 and R27 reverses OmpA dependency (**B**). OmpA-dependent experiment for TraN_V_ (**C**). TraN_L_γ in R27 maintained the OmpA dependency (**D**). **, *P* < 0.01; ****, *P* < 0.0001.

We hypothesized that, like TraN_S_, MPS is mediated by cooperation between the tip domains of TraN_M_ and TraN_L_ in the donor and an outer membrane protein (OMP) in the recipient. To test this hypothesis, we used a selection of *E. coli* OMP deletion mutants from the KEIO collection as recipients (Fig. S1). This has shown that none of the tested mutations affected conjugation efficiency of RA1 (Fig. S1A). In contrast, R27 was conjugated at a lower frequency specifically into a Δ*ompA* recipient (Fig. 8A; Fig. S1B). To validate this, we used the swapped RA1 and R27 tips and the Δ*ompA* recipient, and we also included R27::TraN_V_ and R27::TraN_L_γ. This revealed that conjugation of R27-TraN_M_ and R27::TraN_V_ was OmpA-independent, while conjugation of RA1-TraN_L_ and and R27::TraN_L_γ were OmpA-dependent (Fig. 8B through D). We concluded that TraN_L_α- and TraN_L_γ-mediated MPS is dependent on OmpA, while the partner OMP of TraN_M_ and TraN_V_ remains unknown.

## DISCUSSION

The spread of ARGs is at the heart of the silent AMR pandemic (36). IncA/C and IncH plasmids frequently carry carbapenemases; indeed, a recent study described the spread of an IncH imipenemase-encoding plasmid among diverse Enterobacterales species between 2016 and 2019 across a London regional network (37). Yet, little is known about TraN-mediated MPS and the mechanism of transfer of IncA/C and IncH plasmids.

While observations made in pED208 have shown that a Δ*traN* mutant donor was competent for some F pilus production, as demonstrated by M13K07 phage sensitivity, detection of extracellular TraA, and low-level plasmid transfer (33), we have shown that complete deletion of TraN in pKpQIL, RA1, and R27 resulted in conjugation efficiency below the level of detection. Using our polyclonal pKpQIL pilus antiserum revealed that no pili were seen on the donor containing pKpQIL Δ*traN_S_*. This suggests that while the TraN tip domain in pKpQIL plays a role in MPS, the base domain likely has a structural role in mating pilus biogenesis. However, this feature is not universally conserved, as was demonstrated by the pED208 Δ*traN* mutant (33).

Bioinformatics analysis shows that all the TraN proteins encoded by IncA/C (and IncA/C2) plasmids fall into the TraN_M_ category. While we identified the OMP partners for the TraN_S_ and TraN_L_ variants, the partner of TraN_M_ remains unknown. The TraN_M_ mostly belongs to a single dominant lineage (TraN_M_β), which includes the pNDM-US plasmid, isolated in the USA from *K. pneumoniae* and carrying the *bla*_NDM_ gene (34), while the remainder belong to several diverse lineages (TraN_M_α), including the prototype plasmid RA1. Considering the bias in genome sequencing toward strains recovered from severe clinical cases, this suggests that TraN_M_β is more commonly found in these isolates. While most plasmids encoding TraN_M_ were found in Enterobacterales species, they were also associated with other families, suggesting that IncA/C are broad-host-range plasmids. Our conjugation assays revealed that IncA/C plasmids are conjugated efficiently into the representative Enterobacterales species we used, except into EPEC, possibly due to expression of defense systems (38).

All the TraN proteins expressed by IncH plasmids belong to the TraN_L_ category. AlphaFold3 predictions indicate that, despite sequence and host range variabilities, the TraN_L_ variants share similar structure and use OmpA as a recipient partner. In contrast to TraN_M_, with only one exception, all TraN_L_ proteins have been isolated from Enterobacterales species, suggesting that IncH plasmids mainly circulate specifically within this family. Analysis of the IncHI1B pNDM-MAR plasmid, isolated in the USA from *K. pneumoniae* and carrying the *bla*_NDM_ gene (35), revealed that its TraN belongs to the TraN_L_γ variant. While the TraN_L_γ is naturally found almost exclusively in *K. pneumoniae*, it can mediate efficient DNA transfer into other Enterobacterales species under lab condictions, including EPEC, raising the possibility that additional factors on plasmids endogenously encoding TraN_L_γ allow their replication or maintenance mainly in *Klebsiella* (38).

In addition to the TraN_S_, TraN_M_, and TraN_L_ we also found a small TraN_V_ grouping, unexpectedly encoded by plasmids found in *A. baumannii* in China and South Korea. Structural predictions revealed that the TraN_V_ base domain is extended by two distinct tips. While a chimera of TraN_V_, T1, or T2 on the R27 base resulted in similar conjugation efficiency into *A. baumannii* recipient, only TraN_V_ and T1 were functional when *E. coli* was used as a recipient. This suggests that while the receptor for T1 is shared between the two recipients, the receptor for T2 is only present in *A. baumannii.* Testing conjugation of TraN_V_ into multiple species revealed the highest efficiency into *E. coli, K. pneumoniae,* and EPEC, suggesting that these species could acquire such plasmids. Conjugation into the other species was similar to the level recorded for the Δtip, suggesting that these species have a relatively low likelihood of acquiring TraN_V_-encoding plasmids in the future.

In summary, this study focused on TraN_M_, TraN_L_, and TraN_V_, deepening our understanding of the mechanism of spread of IncA/C, IncH, and the TraN_V_-encoding plasmids in clinically relevant species. Combining our data on TraN-mediated species specificity could potentially be used as predictors for future plasmid transmission.

## MATERIALS AND METHODS

### Identification of TraN homologs and characterization of the associated plasmids

We used “Data set 1” from the Plascad database (6), which contains >14,000 plasmids, which are a mix of conjugative, mobilizable, and non-mobilizable (see attached), to identify publicly-available plasmid sequences possessing the mating pair formation “F” (MPFF) system. We then identified and extracted protein sequences from the associated GenBank files that were annotated as product = “traN” OR “TraN” OR “trhN” OR “TrhN,” or gene = “traN” OR “TraN” OR “trhN” OR “TrhN.” We used Clustal Omega v1.2.4 (39) to align the resulting protein sequences and generate a pairwise similarity matrix. We generated a heatmap showing the pairwise similarities between all TraN proteins of ≥880 aa using the heatmap.2 function from the gplots v.3.2.0 package in RStudio (R v4.4.2).

Plasmid replicon types were identified from the MPFF plasmids using PlasmidFinder (database version 29/11/2021) (40). Carbapenemase genes were identified using AMRFinderPlus v3.10.23, database version 2021-12-21.114 (41). MOB groups and PTU assignments were determined using COPLA v1.0 (42).

### Phylogenetic analysis of TraN homologs

Separate alignments were generated incorporating all TraN protein sequences of ≥880 aa with ≥30% similarity to those from the RA1 and R27 plasmids, respectively. ModelTest-NG v0.2.0 (43) was used to select the most suitable evolutionary model to use in each phylogenetic analysis. RAxML-NG v1.2.0 (44) was used to generate a maximum likelihood phylogenetic tree with 1,000 bootstrap replicates from each alignment. The phylogenetic trees were visualized together with metadata using Microreact (45).

### Bacterial strains and plasmids

The bacterial strains, conjugative plasmids, and mutagenesis vectors used are listed in Tables S1 to S3, respectively. Unless otherwise stated, bacteria were cultured in Lysogeny Broth (LB) at 37°C, 200 r.p.m. When needed, antibiotics were used at the following concentrations: chloramphenicol (30 μg/mL), streptomycin (50 μg/mL), kanamycin (50 μg/mL), gentamicin (10 μg/mL), and tetracycline (10 μg/mL).

### Conjugation assay

All the conjugative plasmids are expressed in trp-*E. coli* MG1655, our conjugative “donor.” Overnight cultures of donor and recipient strains were washed in fresh LB and mixed in a ratio of 10:1 (R27) or 2:1 (RA1). This mixture was further diluted 1 in 25 in fresh LB, and 40 μL was spotted onto LB agar plates. Plates were subcultured for 2–3 h at 37°C before incubation at 25°C (R27) or at 30°C overnight (RA1). The conjugation mixture was collected and resuspended in 1 mL of sterile PBS. The transconjugants were selected by plating the conjugation mixture onto an M9 minimal media agar plate containing the appropriate selection marker (chloramphenicol for R27; tetracycline for RA1). Conjugation frequency was calculated as the ratio of the colony-forming units per mL (CFU/mL) of transconjugants to the CFU/mL of recipients, and the data were log_10_ transformed before statistical analysis.

### Generation of mutants

All genomic mutations were made in *E. coli* MG1655, using a two-step recombination methodology. Mutagenesis vectors were mobilized from *E. coli* CC118λpir into pACBSR-carrying strains through a tri-parental conjugation using the *E. coli* 1047 pRK2013 helper strain. Merodiploid colonies were selected on LB agar containing gentamicin and streptomycin. Selected colonies were grown for at least 4 h in LB supplemented with streptomycin and 0.4% l-arabinose to induce expression of the I-SceI endonuclease from the pACBSR plasmid. Cultures were streaked onto LB agar containing streptomycin and screened for the intended mutations. Mutations in plasmids were introduced using the same methodology.

Mutagenesis vectors were generated by Gibson Assembly (New England Biolabs, E2611L) using the pSEVA612S backbone and were maintained in *E. coli* CC118λpir cells. Site-directed mutagenesis on previously generated vectors was performed according to the Q5 Site-Directed Mutagenesis Kit protocol (New England Biolabs, M0554S). Primers used to generate the mutagenesis vectors and for screening are listed in Table S4. All mutations were confirmed by sequencing (Eurofins).

### Immunofluorescence microscopy of conjugative pili

Overnight cultures were diluted 1 in 20 (vol/vol) in fresh LB and added to glass coverslips placed in a 24-well plate before incubation at 37°C for 1.5 h to allow the bacteria to adhere to the surface of the coverslips. Excess media was removed, and the coverslips were washed with PBS before fixation in 4% PFA for 20 min at room temperature (RT). Fixed samples were washed in PBS and blocked in 2% bovine serum albumin (BSA) in PBS (wt/vol). Samples were washed three times before incubation with anti-pili antibodies (1:100 in 2% BSA/PBS) for 1 h at RT. Samples were washed three times in PBS and incubated with Alexa Fluor 488 conjugated donkey anti-rat IgG antibodies (Jackson Immunoresearch, 1:1,000 in 2% BSA/PBS) for 1 h at RT. Coverslips were washed three times in PBS and incubated with FM4-64 (Invitrogen, 1:100 in water) for 5 min at RT. Following this, coverslips were dried and mounted onto glass slides using VECTASHIELD HardsetTM Antifade Mounting Medium with DAPI (Vector Laboratories) according to the manufacturer’s instructions. Slides were analyzed using a ×100 objective on a Zeiss Axio Observer 7 microscope, and images were processed on Zen 2.3 (Blue Version; Zeiss).

### Generation of TraN AlphaFold models

In the absence of homologous TraN structures, *ab initio* models were generated by AlphaFold3 (46). TraN sequences were submitted to the AlphaFold Colab server with the default settings; the signal peptide was removed from all sequences before modeling. Each structural model was validated by analyzing the confidence score as generated by the predicted local distance difference test (pLDDT). Molecular graphics and superimposition analysis were performed in UCSF ChimeraX-1.8 (47).

### Statistics and reproducibility

All data are representative of 2–4 biological independent repeats. Statistical analyses were performed on GraphPrism 9.5.0 (GraphPad software), and data were checked for normality after Log10 transformation using the Shapiro-Wilk test. Conjugation data were analyzed by repeated measures, one-way ANOVA with Tukey’s or Dunnett’s multiple comparison test, as appropriate (detailed in the figure legends). Where only two recipient strains were being compared, a two-sided paired *t*-test was used. *P* values less than 0.05 were considered significant.

### TraN homologs

TraN sequences were obtained from the following reference plasmids: R27 (accession ID: AF250878), RA1 (accession ID: FJ705807), p-NDM-US (accession ID: CP006661), p-NDM-MAR (accession ID: JN420336), and pABAY10001 (accession ID: MK386682).

## SUPPLEMENTAL MATERIAL

Supplemental figure and tables jb.00536-25-s0001.docx Figure S1 and Tables S1 to S4.

ASM does not own the copyrights to Supplemental Material that may be linked to, or accessed through, an article. The authors have granted ASM a non-exclusive, world-wide license to publish the Supplemental Material files. Please contact the corresponding author directly for reuse.

## References

[1] Kang E, Crouse A, Chevallier L, Pontier SM, Alzahrani A, Silué N, Campbell-Valois F-X, Montagutelli X, Gruenheid S, Malo D. 2018. Enterobacteria and host resistance to infection. Mamm Genome 29:558–576. doi:10.1007/s00335-018-9749-4 29785663

[2] Logan LK, Weinstein RA. 2017. The epidemiology of carbapenem-resistant Enterobacteriaceae: the impact and evolution of a global menace. J Infect Dis 215:S28–S36. doi:10.1093/infdis/jiw282 28375512 PMC5853342

[3] Partridge SR. 2015. Resistance mechanisms in Enterobacteriaceae. Pathology (Phila) 47:276–284. doi:10.1097/PAT.0000000000000237 25764207

[4] Lang AS, Buchan A, Burrus V. 2025. Interactions and evolutionary relationships among bacterial mobile genetic elements. Nat Rev Microbiol 23:423–438. doi:10.1038/s41579-025-01157-y 40069292

[5] Arnold BJ, Huang IT, Hanage WP. 2022. Horizontal gene transfer and adaptive evolution in bacteria. Nat Rev Microbiol 20:206–218. doi:10.1038/s41579-021-00650-4 34773098

[6] Che Y, Yang Y, Xu X, Břinda K, Polz MF, Hanage WP, Zhang T. 2021. Conjugative plasmids interact with insertion sequences to shape the horizontal transfer of antimicrobial resistance genes. Proc Natl Acad Sci USA 118:e2008731118. doi:10.1073/pnas.2008731118 33526659 PMC8017928

[7] Virolle C, Goldlust K, Djermoun S, Bigot S, Lesterlin C. 2020. Plasmid transfer by conjugation in Gram-negative bacteria: from the cellular to the community level. Genes (Basel) 11:1239. doi:10.3390/genes11111239 33105635 PMC7690428

[8] Arutyunov D, Frost LS. 2013. F conjugation: back to the beginning. Plasmid 70:18–32. doi:10.1016/j.plasmid.2013.03.010 23632276

[9] Bondy-Denomy J, Garcia B, Strum S, Du M, Rollins MF, Hidalgo-Reyes Y, Wiedenheft B, Maxwell KL, Davidson AR. 2015. Multiple mechanisms for CRISPR–Cas inhibition by anti-CRISPR proteins. Nature 526:136–139. doi:10.1038/nature15254 26416740 PMC4935067

[10] Frost L, Lee S, Yanchar N, Paranchych W. 1989. finP and fisO mutations in FinP anti-sense RNA suggest a model for FinOP action in the repression of bacterial conjugation by the Flac plasmid JCFLO. Mol Gen Genet 218:152–160. doi:10.1007/BF00330578 2476653

[11] Schröder G, Lanka E. 2003. TraG-like proteins of type IV secretion systems: functional dissection of the multiple activities of TraG (RP4) and TrwB (R388). J Bacteriol 185:4371–4381. doi:10.1128/JB.185.15.4371-4381.2003 12867445 PMC165781

[12] Seddon C, David S, Wong JLC, Ishimoto N, He S, Bradshaw J, Low WW, Frankel G, Beis K. 2025. Cryo-EM structure and evolutionary history of the conjugation surface exclusion protein TraT. Nat Commun 16:659. doi:10.1038/s41467-025-55834-w 39809778 PMC11733297

[13] Costa TRD, Patkowski JB, Macé K, Christie PJ, Waksman G. 2024. Structural and functional diversity of type IV secretion systems. Nat Rev Microbiol 22:170–185. doi:10.1038/s41579-023-00974-3 37814112 PMC11290344

[14] Shintani M, Sanchez ZK, Kimbara K. 2015. Genomics of microbial plasmids: classification and identification based on replication and transfer systems and host taxonomy. Front Microbiol 6:242. doi:10.3389/fmicb.2015.00242 25873913 PMC4379921

[15] Smillie C, Garcillán-Barcia MP, Francia MV, Rocha EPC, de la Cruz F. 2010. Mobility of plasmids. Microbiol Mol Biol Rev 74:434–452. doi:10.1128/MMBR.00020-10 20805406 PMC2937521

[16] Rozwandowicz M, Brouwer MSM, Fischer J, Wagenaar JA, Gonzalez-Zorn B, Guerra B, Mevius DJ, Hordijk J. 2018. Plasmids carrying antimicrobial resistance genes in Enterobacteriaceae. J Antimicrob Chemother 73:1121–1137. doi:10.1093/jac/dkx488 29370371

[17] Gabant P, Newnham P, Taylor D, Couturier M. 1993. Isolation and location on the R27 map of two replicons and an incompatibility determinant specific for IncHI1 plasmids. J Bacteriol 175:7697–7701. doi:10.1128/jb.175.23.7697-7701.1993 8244940 PMC206928

[18] Alonso G, Baptista K, Ngo T, Taylor DE. 2005. Transcriptional organization of the temperature-sensitive transfer system from the IncHI1 plasmid R27. Microbiology (Reading) 151:3563–3573. doi:10.1099/mic.0.28256-0 16272379

[19] Whelan KF, Maher D, Colleran E, Taylor DE. 1994. Genetic and nucleotide sequence analysis of the gene htdA, which regulates conjugal transfer of IncHI plasmids. J Bacteriol 176:2242–2251. doi:10.1128/jb.176.8.2242-2251.1994 7908903 PMC205345

[20] Fricke WF, Welch TJ, McDermott PF, Mammel MK, LeClerc JE, White DG, Cebula TA, Ravel J. 2009. Comparative genomics of the IncA/C multidrug resistance plasmid family. J Bacteriol 191:4750–4757. doi:10.1128/JB.00189-09 19482926 PMC2715731

[21] Bradley DE. 1989. Conjugation system of IncC plasmid RA1, and the interaction of RA1 pili with specific RNA phage C-1. Res Microbiol 140:439–446. doi:10.1016/0923-2508(89)90064-8 2576151

[22] Ishimoto N, He S, Bogdanov M, Smith TK, Frankel G, Beis K. 2025. Phospholipid-independent biogenesis of a functional RP4 conjugation pilus. bioRxiv:2025.06.27.661960. doi:10.1101/2025.06.27.661960 PMC13434634 42310306

[23] Ishimoto N, Wong JLC, He S, Shirran S, Wright-Paramio O, Seddon C, Singh N, Balsalobre C, Sonani RR, Clements A, Egelman EH, Frankel G, Beis K. 2025. Cryo-EM structure of the conjugation H-pilus reveals the cyclic nature of the TrhA pilin. Proc Natl Acad Sci USA 122:e2427228122. doi:10.1073/pnas.2427228122 40244678 PMC12037004

[24] Beltrán L, Torsilieri H, Patkowski JB, Yang JE, Casanova J, Costa TRD, Wright ER, Egelman EH. 2024. The mating pilus of E. coli pED208 acts as a conduit for ssDNA during horizontal gene transfer. mBio 15:e0285723. doi:10.1128/mbio.02857-23 38051116 PMC10790687

[25] Goldlust K, Ducret A, Halte M, Dedieu-Berne A, Erhardt M, Lesterlin C. 2023. The F pilus serves as a conduit for the DNA during conjugation between physically distant bacteria. Proc Natl Acad Sci USA 120:e2310842120. doi:10.1073/pnas.2310842120 37963249 PMC10666033

[26] Fraikin N, Couturier A, Lesterlin C. 2024. The winding journey of conjugative plasmids toward a novel host cell. Curr Opin Microbiol 78:102449. doi:10.1016/j.mib.2024.102449 38432159

[27] Klimke WA, Rypien CD, Klinger B, Kennedy RA, Rodriguez-Maillard JM, Frost LS. 2005. The mating pair stabilization protein, TraN, of the F plasmid is an outer-membrane protein with two regions that are important for its function in conjugation. Microbiology (Reading) 151:3527–3540. doi:10.1099/mic.0.28025-0 16272376

[28] Low WW, Wong JLC, Beltran LC, Seddon C, David S, Kwong H-S, Bizeau T, Wang F, Peña A, Costa TRD, Pham B, Chen M, Egelman EH, Beis K, Frankel G. 2022. Mating pair stabilization mediates bacterial conjugation species specificity. Nat Microbiol 7:1016–1027. doi:10.1038/s41564-022-01146-4 35697796 PMC9246713

[29] Frankel G, David S, Low WW, Seddon C, Wong JLC, Beis K. 2023. Plasmids pick a bacterial partner before committing to conjugation. Nucleic Acids Res 51:8925–8933. doi:10.1093/nar/gkad678 37592747 PMC10516633

[30] Low WW, Seddon C, Beis K, Frankel G. 2023. The interaction of the F-like plasmid-encoded TraN isoforms with their cognate outer membrane receptors. J Bacteriol 205:e0006123. doi:10.1128/jb.00061-23 36988519 PMC10127662

[31] Carattoli A, Villa L, Poirel L, Bonnin RA, Nordmann P. 2012. Evolution of IncA/C bla_CMY-2_-carrying plasmids by acquisition of the bla_NDM-1_ carbapenemase gene. Antimicrob Agents Chemother (Bethesda) 56:783–786. doi:10.1128/AAC.05116-11 PMC3264282 22123704

[32] Galata V, Fehlmann T, Backes C, Keller A. 2019. PLSDB: a resource of complete bacterial plasmids. Nucleic Acids Res 47:D195–D202. doi:10.1093/nar/gky1050 30380090 PMC6323999

[33] Kishida K, Bosserman RE, Harb L, Khara P, Song L, Hu B, Zeng L, Christie PJ. 2022. Contributions of F-specific subunits to the F plasmid-encoded type IV secretion system and F pilus. Mol Microbiol 117:1275–1290. doi:10.1111/mmi.14908 35434837 PMC9359479

[34] Hudson CM, Bent ZW, Meagher RJ, Williams KP. 2014. Resistance determinants and mobile genetic elements of an NDM-1-encoding Klebsiella pneumoniae strain. PLoS One 9:e99209. doi:10.1371/journal.pone.0099209 24905728 PMC4048246

[35] Villa L, Poirel L, Nordmann P, Carta C, Carattoli A. 2012. Complete sequencing of an IncH plasmid carrying the bla_NDM-1_, bla_CTX-M-15_ and qnrB1 genes. J Antimicrob Chemother 67:1645–1650. doi:10.1093/jac/dks114 22511638

[36] Boccabella L, Palma EG, Abenavoli L, Scarlata GGM, Boni M, Ianiro G, Santori P, Tack JF, Scarpellini E. 2024. Post-coronavirus disease 2019 pandemic antimicrobial resistance. Antibiotics (Basel) 13:233. doi:10.3390/antibiotics13030233 38534668 PMC10967554

[37] Wan Y, Myall AC, Boonyasiri A, Bolt F, Ledda A, Mookerjee S, Weiße AY, Getino M, Turton JF, Abbas H, et al.. 2024. Integrated analysis of patient networks and plasmid genomes to investigate a regional, multispecies outbreak of carbapenemase-producing Enterobacterales carrying both bla_IMP_ and mcr-9 genes. J Infect Dis 230:e159–e170. doi:10.1093/infdis/jiae019 39052705 PMC11272044

[38] Oo G, Low WW, Yong M, Stanton TD, Ayuni NN, Bifani P, Wyres KL, Gan Y-H. 2025. Anti-plasmid defense in hypervirulent Klebsiella pneumoniae involves type I-like and type IV restriction modification systems. Emerg Microbes Infect 14:2558877. doi:10.1080/22221751.2025.2558877 40916842 PMC12456054

[39] Sievers F, Higgins DG. 2018. Clustal Omega for making accurate alignments of many protein sequences. Protein Sci 27:135–145. doi:10.1002/pro.3290 28884485 PMC5734385

[40] Carattoli A, Zankari E, García-Fernández A, Voldby Larsen M, Lund O, Villa L, Møller Aarestrup F, Hasman H. 2014. In silico detection and typing of plasmids using PlasmidFinder and plasmid multilocus sequence typing. Antimicrob Agents Chemother 58:3895–3903. doi:10.1128/AAC.02412-14 24777092 PMC4068535

[41] Feldgarden M, Brover V, Gonzalez-Escalona N, Frye JG, Haendiges J, Haft DH, Hoffmann M, Pettengill JB, Prasad AB, Tillman GE, Tyson GH, Klimke W. 2021. AMRFinderPlus and the Reference Gene Catalog facilitate examination of the genomic links among antimicrobial resistance, stress response, and virulence. Sci Rep 11:12728. doi:10.1038/s41598-021-91456-0 34135355 PMC8208984

[42] Redondo-Salvo S, Bartomeus-Peñalver R, Vielva L, Tagg KA, Webb HE, Fernández-López R, de la Cruz F. 2021. COPLA, a taxonomic classifier of plasmids. BMC Bioinformatics 22:390. doi:10.1186/s12859-021-04299-x 34332528 PMC8325299

[43] Darriba D, Posada D, Kozlov AM, Stamatakis A, Morel B, Flouri T. 2020. ModelTest-NG: a new and scalable tool for the selection of DNA and protein evolutionary models. Mol Biol Evol 37:291–294. doi:10.1093/molbev/msz189 31432070 PMC6984357

[44] Kozlov AM, Darriba D, Flouri T, Morel B, Stamatakis A. 2019. RAxML-NG: a fast, scalable and user-friendly tool for maximum likelihood phylogenetic inference. Bioinformatics 35:4453–4455. doi:10.1093/bioinformatics/btz305 31070718 PMC6821337

[45] Argimón S, Abudahab K, Goater RJE, Fedosejev A, Bhai J, Glasner C, Feil EJ, Holden MTG, Yeats CA, Grundmann H, Spratt BG, Aanensen DM. 2016. Microreact: visualizing and sharing data for genomic epidemiology and phylogeography. Microb Genom 2:e000093. doi:10.1099/mgen.0.000093 28348833 PMC5320705

[46] Jumper J, Evans R, Pritzel A, Green T, Figurnov M, Ronneberger O, Tunyasuvunakool K, Bates R, Žídek A, Potapenko A, et al.. 2021. Highly accurate protein structure prediction with AlphaFold. Nature 596:583–589. doi:10.1038/s41586-021-03819-2 34265844 PMC8371605

[47] Meng EC, Goddard TD, Pettersen EF, Couch GS, Pearson ZJ, Morris JH, Ferrin TE. 2023. UCSF ChimeraX: tools for structure building and analysis. Protein Sci 32:e4792. doi:10.1002/pro.4792 37774136 PMC10588335

